# Rheology and Water Absorption Properties of Alginate–Soy Protein Composites

**DOI:** 10.3390/polym13111807

**Published:** 2021-05-31

**Authors:** Estefanía Álvarez-Castillo, José Manuel Aguilar, Carlos Bengoechea, María Luisa López-Castejón, Antonio Guerrero

**Affiliations:** Departamento de Ingeniería Química, Escuela Politécnica Superior, Universidad de Sevilla, Calle Virgen de África, 7, 41011 Sevilla, Spain; jmaguilar@us.es (J.M.A.); cbengoechea@us.es (C.B.); llcastejon@us.es (M.L.L.-C.); aguerrero@us.es (A.G.)

**Keywords:** soy protein, alginate, composite, rheology, water uptake, polymeric matrices

## Abstract

Composite materials based on proteins and carbohydrates normally offer improved water solubility, biodegradability, and biocompatibility, which make them attractive for a wide range of applications. Soy protein isolate (SPI) has shown superabsorbent properties that are useful in fields such as agriculture. Alginate salts (ALG) are linear anionic polysaccharides obtained at a low cost from brown algae, displaying a good enough biocompatibility to be considered for medical applications. As alginates are quite hydrophilic, the exchange of ions from guluronic acid present in its molecular structure with divalent cations, particularly Ca^2+^, may induce its gelation, which would inhibit its solubilization in water. Both biopolymers SPI and ALG were used to produce composites through injection moulding using glycerol (Gly) as a plasticizer. Different biopolymer/plasticizer ratios were employed, and the SPI/ALG ratio within the biopolymer fraction was also varied. Furthermore, composites were immersed in different CaCl_2_ solutions to inhibit the amount of soluble matter loss and to enhance the mechanical properties of the resulting porous matrices. The main goal of the present work was the development and characterization of green porous matrices with inhibited solubility thanks to the gelation of alginate.

## 1. Introduction

It is increasingly frequent to find biopolymers as an integral part of new materials [1,2,3,4], which can be widely used in different applications where they replace traditional plastics. Therefore, these new materials help to reduce the overall environmental footprint, as synthetic plastics have a low degradability. A biopolymer that is extensively used as a raw material in biodegradable materials is soy protein isolate, which is obtained as a by-product from the soy oil industry. This protein is formed by two main globular fractions with different structures and molecular weights, namely glycinin-11S and β-conglycinin-7S proteins. In order to promote a circular economy, this by-product could be reused for different purposes, including the formulation of biodegradable materials. In this sense, several studies have confirmed the feasibility of producing superabsorbent materials [5,6,7] that could potentially be used in different fields, such as horticulture, pharmaceutical, personal care, and textile fields [8,9,10]. A material needs to absorb at least 10 times (1000%) its own weight in water without losing its integrity in order to be classified as a superabsorbent material [9,11]. A three dimensional swollen structure forms simultaneously during the water absorption, resulting in porous matrices when dried, which could be reused again as superabsorbent materials [10].

The porous matrices formed from superabsorbent polymeric materials could be considered for biomedical applications in the field of tissue engineering. A porous structure is always required to promote the blood intrusion and vascularization of nutrients and cells through biomaterials [12]. Polysaccharides such as alginates should also be used to improve biocompatibility, avoiding harmful or toxic effects on the biological system [13]. Thus, including alginate, which shows a good enough biocompatibility and a small inflammatory response in the human body [14], would make these highly porous matrices interesting in some fields such as biomedicine or tissular engineering.

Alginate is a polysaccharide obtained from brown algae, mainly composed of alginic acid and its salts (ALG), which possess a high content in carboxylic groups that confer them hydrophilic and water-binding properties [15,16]. Alginate is a linear negatively charged copolymer of β-d-manuronate (M) and α-l-guluronate (G), for which different sequences result in different molecular weights [17,18]. Residues of manuronic and guluronic acids can be linked either by forming β (1–4) glucosidic links in MM and MG, or by α (1–4) glucosidic links in GG and GM [15]. The gelation of alginate occurs in the presence of multivalent metal ions, as is the case for calcium ions (Ca^2+^), explained by the generally known “egg-box” model for the complexation of aligned chains of negatively charged G with Ca^2+^ [10,19]. Additionally, alginate is an interesting raw material for the food industry and in medicine (e.g., drug delivery tissue engineering) because of its biocompatibility, nontoxicity, and biodegradability [18,20,21,22,23]. Thus, several studies have developed materials containing alginate obtained by different processing strategies, such as casting [17,20,21,24] or capsule formation [23,25,26].

Injection moulding is an extensively employed processing technique in the production of superabsorbent materials from biopolymers [7,27,28,29,30,31,32]. It basically consists of a two-step batch procedure in which a biopolymer/plasticizer blend is obtained, which is subsequently injected into a mould cavity. The viscoelastic properties of the blends are of great importance to define the processing parameters (i.e., cylinder and mould temperatures) for the injection moulding stage [33,34]. Thus, when protein-based blends are used, the glass transition temperature must be overpassed in order to ensure suitable flowability into the mould cavity [35].

Mild moulding temperatures are the main factor for promoting the superabsorbent properties of an injection-moulded material [7,36], as the injection pressure exerts a lesser effect [37]. A plasticizer is needed to ease the processing of blends containing biopolymers as it enhances the plasticity of the materials and facilitates the injection process [38,39]. Glycerol (Gly), the most frequently used plasticizer for proteins and polysaccharides, possesses a high hydrophilicity and is highly miscible with water. Thus, after the immersion of materials in water, Gly is typically transferred from the polymeric matrix onto the aqueous media, resulting in a swollen polymeric structure, where water occupies the pores in which the plasticizer was previously placed [7]. When these swollen structures are freeze-dried, porous matrices are obtained [2,36].

The present manuscript evaluates the feasibility of the production of porous matrices based on soy protein isolates and alginates, as well as its relationship with their rheological properties. Moreover, in order to retain the alginate within the polymeric structure when immersed in water, alginate gelation with Ca^2+^ was induced through the immersion of the injection moulded materials in aqueous solutions of calcium chloride (CaCl_2_).

## 2. Materials and Methods

### 2.1. Materials

Soy protein isolate (SPI) with the trade name SUPRO 500E manufactured by Dupont (Wilmington, DE, USA) was kindly provided by PROANDA S.A. (Sevilla, Spain) and was used as a raw material. Its protein content was 91.8 wt%, which was calculated by quadruplicate, multiplying the *n*% with a Kjeldahl factor of 6.25. The nitrogen content was determined using a LECO CHNS-932 nitrogen microanalyzer (Leco Corporation, St. Joseph, MI, USA). The lipid content and moisture of the SPI were 1.0% and 6.0 wt%, respectively, whereas its ash content was ca. 5.0 wt%. The ALG used was alginic acid sodium salt from brown algae was purchased from Sigma Aldrich (Darmstadt, Germany). Glycerol (Gly) from Panreac Química S.A. (Barcelona, Spain) was employed as a plasticizer to improve the processability of the blends used to obtain the composites. Injection moulded composites were also immersed in solutions of calcium chloride, which were provided by Panreac Química S.A.

### 2.2. Sample Preparation

In the first stage, SPI was mixed with ALG and Gly at different weight ratios, as shown in Table 1. Approximately, a total mass of 65 g was mixed in a two-blade counter-rotating batch mixer Haake Polylab QC (ThermoHaake, Karlsruhe, Germany), at room temperature and 50 rpm for 30 min. This mixer allowed for recording the evolution of the torque and temperature during the blending.

The subsequent blends were submitted to an injection moulding stage carried out in a miniJet Piston Injection Moulding System (ThermoHaake, Karlsruhe, Germany), which gave rise to rectangular-shaped samples (60 × 10 × 1 mm^3^); the cylinder and mould temperatures were set at 50 and 70 °C, respectively; the injection pressure used was equal to 500 bar for 20 s; and the holding pressure applied was 500 bar for 200 s.

Porous polymeric matrices were obtained after being subjected to dehydrothermal treatment (DHT), which consisted of storing the injection moulded composites in an oven at 50 °C for 24 h. Subsequently, the samples were immersed in water for 24 h and swollen specimens were finally freeze-dried at −80 °C and 0.01 mbar in a LyoQuest freeze-dryer (Telstar Technologies, Barcelona, Spain).

### 2.3. Methods

#### 2.3.1. Dynamic Mechanical Analysis (DMA)

The (SPI + ALG)/Gly blends obtained after mixing were rheologically characterized through dynamic mechanical thermal analysis (DMTA) under compression mode in an RSA-3 rheometer (TA Instruments, New Castle, DE, USA). DMTA tests were performed from 25 to 140 °C at a heating rate of 5 °C/min, a compression strain of 1 Hz was applied, and an 8 mm diameter-cylinder geometry was employed.

The (SPI + ALG)/Gly composites resulting from the injection moulding stage were also rheologically tested in the RSA-3 rheometer (TA Instruments, New Castle, DE, USA) through the DMTA tests in the tensile mode. In this case, the frequency sweep tests were carried out from 0.02 to 20 Hz.

The viscoelasticity of the matrices developed after freeze-drying was characterized through frequency sweep tests in compression mode, using an RSA-3 rheometer (TA Instruments, New Castle, DE, USA). The frequency range used was from 0.02 to 20 Hz and the matrices were placed in an 8 mm diameter cylinder geometry.

All of the DMA tests were carried out within the linear viscoelastic region (LVR). Accordingly, the strain sweep tests were performed in advance so as to identify a suitable strain for the linear viscoelasticity characterization of blends, composites, or matrices.

#### 2.3.2. Tensile Tests until Break

The extensional tests were performed using a constant uniaxial extension with a rate of 0.01 mm/s until the failure of the rectangular geometries in an RSA3 rheometer (TA Instruments, New Castle, DE, USA). From this, the main mechanical properties were obtained: Young’s modulus (E), maximum tensile strength (σ_max_), and maximum strain (ε_max_) of the injection moulded composites. These tensile tests were carried out at room temperature and at least six replicates of every sample were performed in order to obtain reliable values for the mechanical parameters.

#### 2.3.3. Water Uptake Capacity

The water uptake of the injection moulded composites was estimated following the methodology described previously [36]. The composite was initially weighed after storage at 50 ± 2 °C, during 24 h (w_1_); then, the dried probe was immersed in water for 24 h and was subsequently weighted (w_2_); and, finally, the swollen sample was stored again in the oven at 50 ± 2 °C for 24 h (w_3_). The water uptake capacity (WUC) and soluble material loss (SML) were calculated as follows:

WUC (%) = (w_2_ − w_3_)/w_3_·100 (1)

SML (%) = (w_1_ − w_3_)/w_1_·100 (2)

#### 2.3.4. Statistical Analysis

In the present manuscript, all of the measurements were performed at least in triplicate. A statistical analysis was performed using the statistical package SPSS 18 through t-test and one-way analysis of variance (ANOVA; *p* ≤ 0.05) performed in Statgraphics software (The Plains, VA, USA). The mean values ± standard deviations were used to express uncertainly, which was determined for all of the calculated parameters.

## 3. Results

### 3.1. Mixing Stage

**Figure 1:**
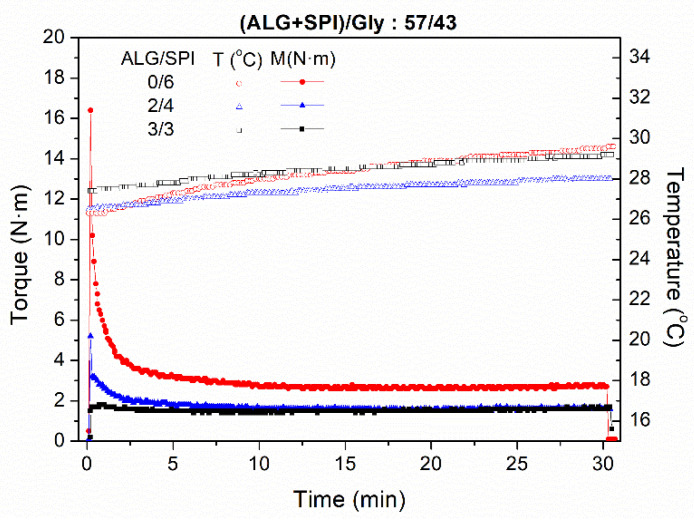
Torque and temperature evolution within the mixer cavity during the mixing stage for alginate (ALG) + soy protein isolate (SPI)/glycerol blends with different ALG/SPI ratios.

Figure 1 shows the evolution of torque obtained after mixing specific amounts of the selected ingredients (SPI, ALG, and Gly) inside the two-blade counter-rotating batch mixer, which is typical for these kinds of polymeric blends [39]. As can be seen, the achievement of a sudden maximum value at the very beginning of the stage is followed by an exponential decrease, which eventually leads to a steady value. As the ALG/SPI ratio increases, a general decrease in torque is observed, and the initial maximum is practically unnoticeable for the sample containing the greatest amount of alginate (blend 3/3). This decrease could be related to the differences between the density of the SPI and ALG flours used, which means that for the same weight of the sample (i.e., 65 g), the volume filled in the mixer is lower because the amount of ALG is higher [38]. Additionally, the samples with ALG in their composition reach their steady values at shorter times, being around 3 min for the sample with ALG/SPI ratio 2/4, and nearly 0 min for those with a ratio of 3/3. Furthermore, the sample that did not contain any ALG (blend 0/6) also showed the highest steady value.

Regarding the temperature evolution with the mixing time inside the mixer cavities, no significant changes were perceived between the different samples, with the maximum increase of temperature being around 2 °C during the whole mixing stage for all of the formulations studied. This small increase would account for the inexistence of important reticulation or crosslinking interactions between the biopolymers during the mixing stage, as no thermal dissipation of mechanical energy was recorded [32]. However, the plasticization energy, which is related to the integral under the torque–time curve, was clearly reduced when SPI is replaced by ALG. This difference can be attributed to the initial step of the mixing process (i.e., when the peak torque is reached) at which the plasticizer (glycerol) diffuses inside the SPI and ALG particles. Both biopolymer particles undergo a fast swelling, absorbing the plasticizer, whereas a solid-like state is formed until a maximum torque is reached. Then, the torque decreases as the SPI and ALG domains are blended and homogenized, eventually reaching a steady state plateau. This reduction in the energy required for plasticization indicates an enhancement of the mixing process when the ALG proportion in the blend is increased. Cordoba et al., 2008 [40], found a similar effect for starch/alginate/glycerol blends. These authors explained the role of alginate in terms of a higher solubility of alginate in glycerol, which can eventually develop an increase in the plasticizing efficiency of Gly, leading to a lower torque peak.

As the thermal response of every sample is similar regardless of the ALG/SPI ratio, neither major protein–protein nor protein–polysaccharide interactions were expected to occur during mixing. It has been reported that different plasticizers or protein sources could promote a noteworthy reticulation of polymeric blends within the mixer, resulting in an important increase of temperature during the mixing stage [39,41].

### 3.2. Rheological Characterization of Composite Blends

**Figure 2:**
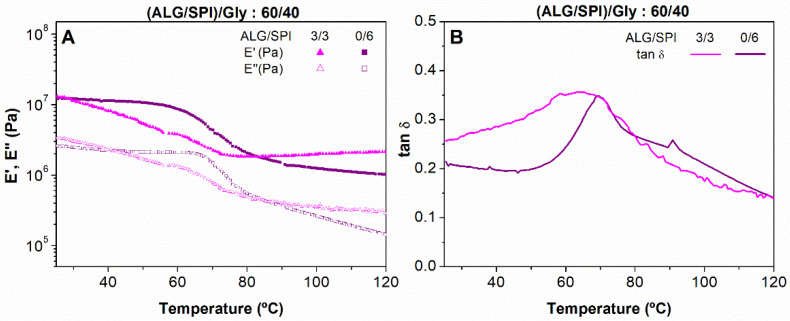
Evolution of the viscoelastic moduli (**A**) and loss tangent (**B**) with temperature of alginate + soy protein isolate/glycerol blends with ALG/SPI ratios: 0/6, 2/4, and 3/3, maintaining the ALG + SPI/Gly ratio of 60/40.

The influence of alginate on the rheology of the samples was first assessed by comparing the samples with an identical biopolymer ALG + SPI/plasticizer (Gly) ratio, and different biopolymer composition (ALG/SPI: 3/3 and 0/6) through DMTA (dynamic mechanical thermal analysis). As can be observed in Figure 2, both samples showed a predominant elastic behavior, which was identified by the higher values of the elastic moduli (E’) when compared with those of the viscous moduli (E’’), resulting in loss tangent values (tan δ) lower than the unity (Figure 2B), as this parameter is related to both moduli (E’’/E’). Furthermore, the viscoelastic moduli decreased as the samples were heated during the test for the whole temperature range studied, showing a thermoplastic behavior, which has previously been reported for similar SPI/Gly systems [33,37,42]. This decrease would be linked to the increased mobility of the polymeric chains promoted by temperature. In this gradual decrease of the viscoelastic parameters, three different sections can be distinguished. (i) The first zone, where a moderate decrease corresponding to the glassy state of the mixture was observed, which was followed by a more dramatic decrease in both moduli. (ii) The second section was commonly related to the glass transition temperature (T_g_) where a maximum in tan δ was observed [39,43]. (iii) Finally, a steady value with lower values in both viscoelastic moduli was raised in the third zone, which correspondsed to a rubbery state.

When the evolution of the viscoelastic moduli was assessed (Figure 2A), it was clear that the presence of ALG did not exert any strong influence on the viscoelastic moduli of these kinds of blends at room temperature (25 °C). However, the presence of ALG seemed to modify the dependence on temperature for the range of temperatures studied. This is related to the fact that the ALG/SPI: 3/3 sample underwent a smaller drop from the initial viscoelastic moduli (83.63%) compared with the blend not containing ALG (blend 0/6) (87.42%). It could be also observed that the presence of alginate (3/3 blend) produced a reduction in section I (or shifting towards lower temperatures). From 80 °C onwards, a tendency towards a rubbery plateau could be noticed, but without reaching a stable value.

Regarding the evolution of tan δ (Figure 2B), a narrow peak could be distinguished at 76 °C for mixture 0/6, which could be attributed to the glass transition of the plasticized SPI. However, the sample containing the 3/3 mixture of biopolymers also showed a simple peak, but it was much broader, between 60–65 °C. The existence of this broad single peak for tan δ might be regarded as the overlap of two glass transitions, one for the plasticized SPI and the other one, obtained at a lower temperature, for the plasticized ALG biopolymer. In fact, the evolution of E’ at a low temperature is quite different for the systems containing the two biopolymers, which reflected the occurrence of two overlapped thermal events. The reduction in the glass transition of protein as a consequence of the plasticizing effect of glycerol and its effect on their viscoelastic properties has been extensively described in previous studies [33,34,44,45]. As reported by Gao et al. (2017) [46], glycerol is also able to induce a remarkable plasticizing effect on sodium alginate. However, this effect could also indicate a certain degree of compatibility between SPI and ALG [44], which would be favoured by the presence of Gly as, as mentioned above, the combination of ALG and Gly could promote plasticizing efficiency on SPI. In other words, a dual role could be ascribed to Gly, acting as a plasticizer and as a compatibilization agent.

**Figure 3:**
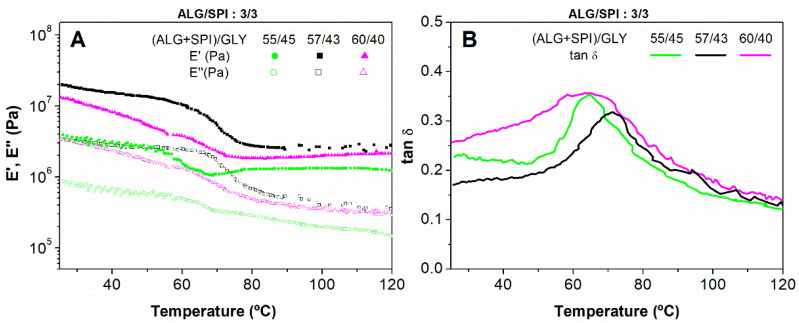
Evolution of the viscoelastic moduli (**A**) and loss tangent (**B**) with temperatures for alginate + soy protein isolate/glycerol blends with different ratios: 55/45, 57/43, and 60/40, maintaining the ALG/SPI ratio of 3/3.

The effect of temperature on the viscoelastic moduli of blends with an identical ALG/SPI ratio (3/3) but different ALG + SPI/Gly ratios are shown in Figure 3. All of the studied samples also showed a predominantly elastic character with a thermoplastic behavior, as a continuous decrease was observed for both viscoelastic moduli as the temperature was increased. It can be noticed that at a low temperature, ALG-containing samples always showed a slight reduction in viscoelastic moduli compared with the system containing SPI as the only biopolymer (0/6), which showed a glass-like region, as seen in Figure 2. These results suggest that the glass state was shifted towards lower temperatures because of the presence of ALG, which is also favoured by increasing the proportion of the plasticizer (Figure 3).

Samples with higher ALG + SPI/Gly ratios (57/43 and 60/40) showed similar E’ and E’’ values at the beginning and the end of the DMTA test, but the values at the intermediate temperatures were reasonably different. Otherwise, the 55/45 sample showed noteworthy lower values for E’ and E’’ for the whole range of temperatures, which might have been induced by the higher quantity of the plasticizer, which weakened the interactions between polymeric chains [37]. Additionally, a temperature rise seemed to cause a slight increase in the elastic moduli after the initial decay for the systems with ALG + SPI/Gly 55/45 and 60/40. This could be related to some kind of protein aggregation, as this has been already reported for analogous protein systems containing albumin, especially when the albumin content is important [36,38,47].

All of the samples displayed a similar evolution for tan δ with temperature (Figure 3B), showing a remarkable maximum that defined the T_g_ of the samples. Thus, the 55/45 and 60/40 blends showed similar values of T_g_ around 62 °C, while the intermediate sample (57/43) showed a higher one, at around 73 °C.

These results suggest that the 57/43 sample possessed a greater thermal stability than the other ones. Hence, this blend was selected to assess the influence of the alginate presence in the injection moulded composites studied in the present manuscript. Furthermore, as mentioned above, the glass transition temperature needed to be taken into account in order to select the processing parameters (i.e., cylinder and mould temperatures).

### 3.3. Rheological Characterization of Injection Moulded Composites

**Figure 4:**
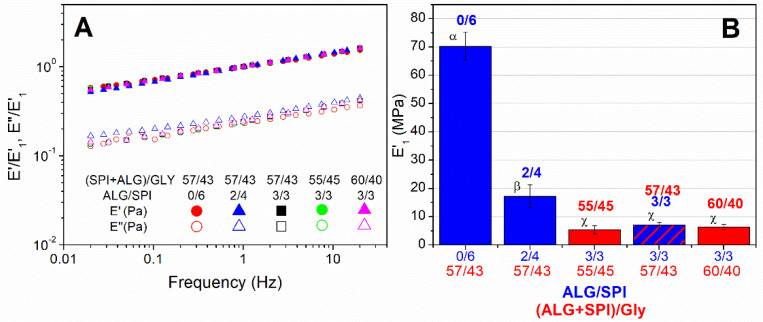
Normalized mechanical spectra of alginate + soy protein isolate/glycerol composites with ratios of: 55/45, 57/43, and 60/40, keeping the ALG/SPI ratio at 3/3, and with ALG/SPI ratios: 0/6, 2/4, and 3/3, keeping the ALG + SPI/Gly ratio at 57/43 (**A**). The elastic moduli of the samples obtained at 1 Hz (**B**). Greek letters indicate that average values are statistically different (*p* < 0.05).

Figure 4A shows the normalized viscoelastic spectra at room temperature for the injection moulded composites with different ALG/SPI and ALG + SPI/Gly ratios studied in the present manuscript. The normalization of the viscoelastic spectra was carried out by dividing the viscoelastic moduli of each system by their corresponding value of the storage modulus at 1Hz (E’_1_). Through this normalization procedure, the displacement of all of the spectra was carried out in the *Y*-axis, matching all of the same master spectrum, as may be observed in Figure 4A. This means that every sample exhibits a prevalent elastic behavior at all of the frequencies tested (E’ > E’’), showing the same dependence on frequency. Only the loss modulus of sample ALG/SPI 2/4 slightly deviated from the master (or normalized) spectrum at a low frequency.

As may be observed in Figure 4B, the ALG/SPI ratio exerts a strong effect on E’_1_ and, consequently, on the viscoelastic moduli during the whole mechanical spectra. Thus, E’_1_ undergoes a dramatic decrease as SPI is being replaced by ALG, giving rise to some relaxation of the protein–protein interactions [36]. Based on this fact, it could be concluded that SPI exhibits a higher structuring capacity than ALG, reducing the viscoelastic moduli by almost 10 times when the ALG/Gly ratio varied from 0/6 to 3/3. In addition, some authors have already reported that alginate may act synergistically with glycerol in starch-based blends, increasing the plasticization degree and efficiency [40]. As for the effect of the biopolymer/Gly ratio, no clear evolution with the plasticizer content could be distinguished, as no significant differences were observed in parameter E’_1_. Previous results carried out with 0/6 ALG/SPI showed a significant plasticizing effect when Gly was increased in a wider proportion than the one tested in this study. However, when using the same SPI/Gly ratio (i.e., 55/45 to 60/40), this effect only led to slight differences [37]. In any case, the results obtained suggest that the presence of ALG contributes to compensating the reduction in Gly.

Henceforth, in view of the non-significant effect found for the ALG + SPI/Gly ratio, this study will be focused on the effect of the ALG/SPI ratio.

### 3.4. Mechanical Properties of Bioplastic Samples

**Figure 5:**
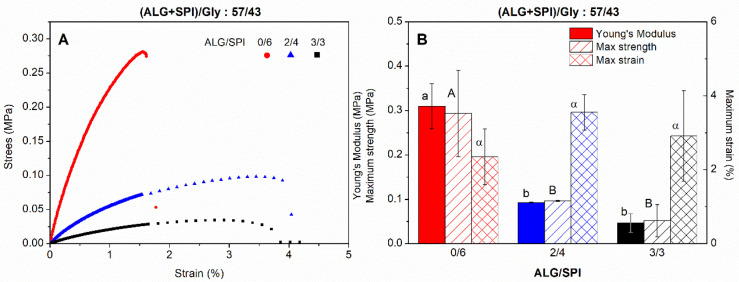
Strain–stress curves (**A**) and main mechanical parameters (**B**) of alginate + soy protein isolate/glycerol composites with different ALG/SPI ratios (0/6, 2/4, and 3/3), keeping the ALG + SPI/Gly ratio at 57/43. Lower-case, upper-case, and Greek letters indicate that average values are statistically different (*p* < 0.05).

The ALG + SPI/Gly composite samples at a 57/43 ratio were tested through uniaxial tension tests as a function of the ALG/SPI proportion. The strain–stress curves obtained showed remarkable quantitative differences between samples as the ALG/SPI ratio increased. However, all of the curves displayed in Figure 5A were qualitatively similar, showing an initial linear dependence between strain and stress, which is commonly known as linear elastic deformation, from which Young’s modulus (E) could be estimated. Then, a slight reduction in the slope was observed, depicted by a small stress causing a noteworthy strain as the samples were deformed, finally leading to the rupture of the material, where the maximum strain (ε_max_) and stress (σ_max_) could be calculated.

All of these mechanical parameters are plotted in Figure 5B, where it can be easily seen that the amount of ALG in the bioplastic affects their mechanical properties, as E notably reduced as the amount of ALG increased in the formulation. Thus, E was 0.31 ± 0.05 MPa for sample 0/6, while it was 0.093 ± 0.01 MPa for sample 2/4, which was further reduced up to 0.047 ± 0.02 MPa for composite 3/3. The evolution of the maximum strength followed an analogous sequence as the alginate content increased, from 0.29 ± 0.09 MPa to one-third of this value. On the other hand, the maximum strain slightly increased with the presence of ALG. Briefly, the sample exclusively composed of SPI showed a stronger but more fragile behavior than the ALG + SPI composites. Similar results were found when the porcine plasma protein was introduced in analogous SPI-based materials [38]. Thus, the reduction in E may again be associated with the above mentioned higher structuring capacity of SPI.

### 3.5. Water Uptake Capacity and Soluble Matter Loss

**Figure 6:**
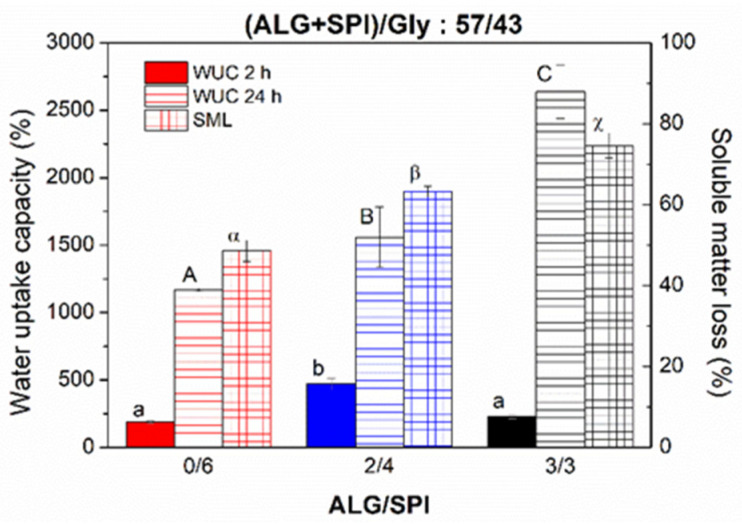
Water uptake capacity after water immersion for 2 and 24 h and soluble matter loss of alginate + soy protein isolate/glycerol composites with ALG/SPI ratios: 0/6, 2/4, and 3/3, maintaining the ALG/SPI/Gly ratio: 57/43. Lower-case, upper-case, and Greek letters indicate that average values are statistically different (*p* < 0.05).

The water uptake capacity (WUC) of the injection-moulded-composite samples with different values for the ALG/SPI ratio were studied after water immersion for 2 and 24 h (Figure 6). After 2 h of water immersion, WUC seemed not to have a direct dependence on the biopolymer ratio, as the WUC initially increased when the ALG/SPI ratio was 2/4. However, no further evolution was observed for the 3/3 ratio. In contrast, after immersion for 24 h, a direct dependence existed between WUC and ALG/SPI, depicted by a clear rise in WUC, as the amount of ALG was higher in the sample (i.e., WUC increased from 1170 ± 110% to 2367 ± 201% when the ALG/SPI ratio shifted from 0/6 to 3/3). In these terms, all of the samples studied in this manuscript showed values of WUC higher than 1000%, which would point out its potential application in the field of superabsorbent materials [2,32]. On the other hand, changes in the soluble matter loss (SML) were also remarkably dependent on ALG/SPI, increasing from 48.6 ± 2.6% to 63.3 ± 1.3% and 74.6 ± 2.8% as the ALG/SPI ratio became higher. Even if Gly is released to the aqueous media during immersion, as suggested by its important hydrophilic properties, these SML values would always overpass the quantity of Gly used in the formulation of probes. In fact, the SML values were similar to the amount of ALG + Gly (43, 62, and 72.5%, respectively), indicating that ALG (and even a small quantity of SPI) could also be released into water. The high value of SML corresponding to sample 3/3 led to a weakening of the polymer network, giving rise to a loss of physical integrity, which made it difficult to handle.

**Figure 7:**
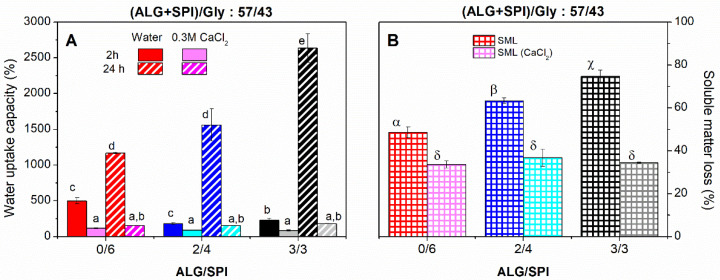
Water uptake capacity after 2 and 24 h of water or CaCl_2_ solution (0.3 M) immersion (**A**) and soluble matter loss (**B**) of alginate + soy protein isolate/glycerol composites with ALG/SPI ratios: 0/6, 2/4, and 3/3, maintaining the ALG + SPI/Gly ratio: 57/43. Lower-case, upper-case, and Greek letters indicate that average values are statistically different (*p* < 0.05).

In order to avoid the solubilization of ALG and the excessive SML observed in Figure 6, samples were introduced in a 0.3 M solution of CaCl_2_ to promote the so-called “egg-box” arrangement within the polymeric framework, which would allow for the fixation of ALG. The results of WUC after 2 and 24 h and SML after immersion in a CaCl_2_ solution are shown in Figure 7. The immersion in the CaCl_2_ solution led to a clear reduction in SML, as observed in Figure 7B, resulting in a similar value for all of the samples (around 37%). Interestingly, this value was even lower than the quantity of Gly employed in each blend. This fact would indicate that the interaction of ALG with Ca^2+^ partially sealed the surface of the matrix, avoiding the complete release of Gly. As a counterpart, the immersion of the sample in a CaCl_2_ solution strongly hindered the water absorption capacity for all of the cases (Figure 7A), even if SPI was the only biopolymer present in the sample. This fact may be associated with the following two different mechanisms: (i) the ions dissolved in the immersion media increase the ionic strength that hinders the swelling [48,49], and (ii) the presence of CaCl_2_ may strengthen the SPI structure [50]. On the other hand, when focused on the sample that previously presented the highest WUC (3/3), an extreme reduction in WUC of around 14 times (from 2637 ± 201 to 180 ± 2%) was observed after a 24 h immersion in the CaCl_2_ solution (0.3 M). This dramatic reduction could be explained on the basis of the formation of the “egg-box” structure after the formation of the complex between Ca^2+^ and ALG [19]. This rearrangement caused crosslinking and inhibited the formation of pores within the matrix, and consequently also hindered the diffusion of water through the structure and its swelling. Likewise, other studies reported an important decrease in WUC when thermal or chemical crosslinking was induced [7,51]. It could also be seen that the sample without ALG (0/6) absorbed a lower amount of water, which might be caused by the presence of salts in the medium that increased the ionic strength of the aqueous media.

### 3.6. Rheological Characterization of Freeze-Dried Matrices After Swelling

**Figure 8:**
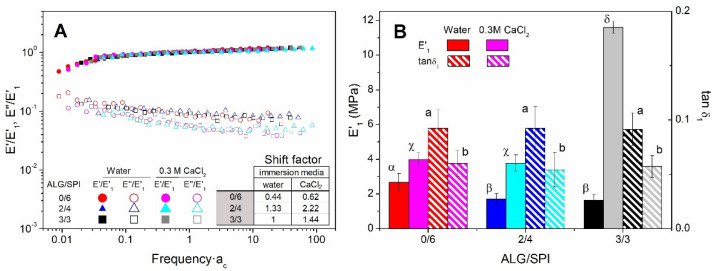
Factorized mechanical spectra of the viscoelastic moduli of alginate + soy protein isolate/glycerol matrices with ALG/SPI ratios: 0/6, 2/4, and 3/3, keeping the ALG + SPI/Gly ratio at 57/43. Freeze-dried matrices were obtained after immersion in water or a 0.3 M CaCl_2_ solution (**A**). Elastic moduli and loss tangent of samples obtained at 1 Hz (**B**). Lower-case, upper-case, and Greek letters indicate that average values are statistically different (*p* < 0.05).

Figure 8A shows the factorized mechanical spectra obtained through the frequency sweep tests of the samples subjected to immersion for 24 h (either in water or CaCl_2_ solution) and subsequently freeze-dried. All of the mechanical spectra were first normalized with E’_1_, following the above-mentioned procedure. Then, a time-composition factor (a_c_) was used to shift the frequency axis for some of the systems. The values of the shift factor are included as an inset of Figure 8A. The lowest values corresponded to the matrices containing SPI as the only biopolymer (0/6). It is worth mentioning that the master curve for E’ was unique, regardless of the immersion medium used, whereas E” matched the curve for water and another for CaCl_2_ solution.

As observed in Figure 8B, when the immersion media was water, the incorporation of ALG produced a decrease in the viscoelastic moduli of the freeze-dried matrices, although no further reduction could be found when ALG was subsequently increased. This matrix weakening could be associated with the following two effects: (i) the lower structuring capacity of ALG compared with SPI, and (ii) the higher porosity of the matrix, as alginate was expected to be essentially removed.

On the other hand, if the freeze-dried matrices were obtained after swelling in the CaCl_2_ solution, an initial replacement of SPI by ALG did not exert any significant effect on E’_1_. It seems in this case that the expected weakening associated with the replacement of SPI might have been overcome by the building up effect caused by the formation of the “egg-box” arrangement in the presence of Ca^2+^. However, when the ALG/SPI ratio increased, a remarkable structural reinforcement (reflected by parameter E’_1_) was observed, as the second effect became clearly dominant. It can also be noticed that the loss tangent at 1 Hz (tan δ_1_) did not significantly depend on the ALG/SPI ratio, regardless of the immersion medium used.

Thus, the reinforcement suffered by the sample that only possessed SPI as a biopolymer (0/6) when immersed in the salt solution was around 1.5 times that obtained when immersed in water (from 2.67 to 3.98 MPa). This interaction between ALG and Ca^2+^ became even more remarkable for the sample with the highest content in ALG (3/3), which was reinforced by around seven times (from 1.64 to 1.16 MPa). As for the tan δ_1_ values, it can be observed that a significant drop took place when a CaCl_2_ solution was used as the immersion medium instead of water. This effect was consistent with the predominant strengthening effect driven by the “egg-box” arrangement.

## 4. Conclusions

The addition of alginate into the formulation of glycerol-plasticized soy protein isolate blends and injection-molded bioplastic matrices is studied. The behavior of the blends indicates that a replacement of soy protein with alginate leads to an enhancement of the mixing process as the plasticization energy decreases, which was previously attributed to an enhancement of the plasticizing efficiency. However, the addition of alginate does not alter their typical thermoplastic behavior. Thus, composite materials obtained when the alginate and soy protein isolate are mixed and injection-moulded still display a decrease of viscoelastic moduli when heated. However, a shift of the glassy plateau towards lower temperatures, together with a decrease of both moduli, are detected when the soy protein is replaced by alginate in the formulation. An enhancement of the plasticizing role of glycerol can be then attributed to alginate. In other words, the plasticizing effect of glycerol for the soy protein and alginate eventually brings about a certain degree of compatibility between both biopolymers. This effect is also reflected on the mechanical properties of the composites—the samples form a fragile material with higher values for Young’s modulus and maximum stress when alginate is absent. However, when the amount of alginate is increased, they tend to be more easily deformable.

The presence of alginate also promotes a higher water uptake capacity when immersed in water. During immersion into water, all glycerol and alginate present in the samples are lost, leading to an increase in the soluble matter loss. However, the presence of Ca^2+^ ions in the immersion medium always results in a reduction in both the water uptake capacity and the soluble matter loss. This is especially noticeable for composite samples containing alginate as an “egg-box” arrangement, because the interaction between alginate and Ca^2+^ ions is induced, hindering the swelling capacity. In these cases, lower soluble matter loss values indicate that alginate remains in the composite sample because of the rearrangement, and only some glycerol is solubilized. Thus, the reinforcement of composite matrices after immersion in a CaCl_2_ solution is apparent through the rheological characterization of the matrices obtained after freeze-drying swollen samples, as higher values of the elastic modulus and lower values of the loss tangent are detected.

Alginate clearly affects the properties of soy protein isolate materials, either resulting in a reinforcement or a softening of the composite in the presence or absence of calcium ions, respectively. Future research should focus on testing the feasibility of these composites as biomaterials for tissue engineering.

## Figures and Tables

**Table 1:** Compositions of the different studied Alginic acid sodium salt (ALG) + soy protein isolate (SPI)/glycerol (Gly) blends.

(ALG + SPI)/Gly	ALG/SPI	wt%		
		SPI	ALG	Gly
60/40	3/3	30	30	40
57/43	3/3	28.5	28.5	43
55/45	3/3	27.5	27.5	45
57/43	0/6	57	0	43
57/43	2/4	38	19	43

## Data Availability

All of the results shown in the manuscript are available upon request from the corresponding author.
